# Tips for avoiding common mistakes in out-of-hospital diagnosis of carbon monoxide poisoning

**DOI:** 10.1186/s44158-022-00041-y

**Published:** 2022-04-02

**Authors:** G. Fucili, M. Brauzzi

**Affiliations:** Sant’Anna School of Advanced Studies, PISA, Italy

**Keywords:** Carbon monoxide poisoning, Diagnosis, Hyperbaric oxygen

## Abstract

Acute carbon monoxide poisoning is the leading cause of intoxication from exogenous substances in the world. It is also a major cause of morbidity and mortality due to poisoning in the USA. In the USA, it determines to 50,000 visits per year in emergency departments with a mortality ranging from 1 to 3%. Although prevalence and incidence data reveal the large impact of carbon monoxide poisoning on public health, some studies have shown that errors in its diagnosis have a high incidence (30%) and that awareness campaigns have allowed the reduction of the same to 5%. In addition, many diagnostic and/or therapeutic errors were found both in small first aid situations and in the context of rescue units belonging to prestigious hospitals. To formulate a diagnosis, the collection of clues from the environment in which the patient is found is essential. Especially when the routine use of environmental gas detectors or handheld CO-oximeters is not possible, the emergency doctor, in addition to concentrating on the clinical presentation of the case, will have to give a quick overview of the patient and his environment. In addition to age, sex, and already known comorbidities, it is not irrelevant to evaluate socio-economic and cultural characteristics, hygiene conditions, habits, etc.

The purpose of this study is to provide useful information to the doctor who comes first to the site of intoxication to reduce diagnostic and therapeutic errors in the pre- and intra-hospital phase as much as possible.

## Introduction

Carbon monoxide poisoning is one of the leading causes of morbidity and mortality due to poisoning in the USA. In the USA, it determines to 50,000 visits per year in emergency departments with a mortality ranging from 1 to 3% [1]. The relationship between the severity of clinical signs and symptoms of acute carbon monoxide poisoning and COHb levels is not well correlated. The poor correlation may be due to the length of time elapsed between cessation of exposure and measurement of COHb levels or to effects of supplemental oxygen treatment prior to COHb measurement.

Although prevalence and incidence data reveal the large impact of carbon monoxide poisoning on public health, some studies have shown that errors in its diagnosis have a high incidence (30%) and that awareness campaigns have allowed the reduction of the same to 5% [2]. In addition, many diagnostic and/or therapeutic errors were found both in small first aid situations and in the context of rescue units belonging to prestigious hospitals [3].

To formulate a diagnosis, the collection of clues from the environment in which the patient is found is essential. Especially when the routine use of environmental gas detectors or handheld CO-oximeters is not possible, the emergency doctor, in addition to concentrating on the clinical presentation of the case, will have to give a quick overview of the patient and his environment. In addition to age, sex, and already known comorbidities, it is not irrelevant to evaluate socio-economic and cultural characteristics, hygiene conditions, habits, etc. Together with a series of specific circumstantial elements, all these aspects will contribute to the formulation of the right diagnosis [4].

Worldwide, the incidence of CO poisoning does not differ between the sexes, while mortality is double for men. Incidence peaks have been reported between 0 and 14 years old and between 20 and 39 years old, respectively. The percentage of patients who died increases with age and grows sharply from 80 years old upwards. Comorbidities and older ages increase mortality.

Intoxication is also directly related to the socio-demographic index (SDI) and mortality is 2.1 to 3.6 times higher in countries with middle to high SDI. However, this data is still being studied and presents objective difficulty when searching for adequate information from economically disadvantaged countries [5].

The purpose of this review is to provide practical and useful tips to decrease diagnostic and therapeutic errors in the pre- and intra-hospital setting.

## Methods

Internet research was carried out using the PubMed electronic database by combining the terms “carbon monoxide poisoning,” “misdiagnosis,” and selecting articles that had one or both of these terms in the title, abstract, or body of the article. Other sources have been identified using articles already in our possession. S2K guidelines on diagnosis and treatment of carbon monoxide poisoning were used [6].

### Epidemiology in brief

Exposure of the general population to carbon monoxide occurs through inhalation of outdoor and indoor air. Populations living in urban areas with heavy vehicular traffic or stationary sources such as petroleum refineries, gas and coal burning power plants, petrochemical plants, and coke oven plants are more likely to be exposed to higher levels of carbon monoxide from ambient outdoor air.

There is a strong association between self-induced carbon monoxide poisoning due to attempts at self-harm and psychiatric pathologies, as well as between suicide and alcohol or drug abuse.

A recent retrospective study of the Danish population discovered that patients already dead at the scene more frequently had abused alcohol or drugs than those who arrived in hospital alive [7].

Atmospheric conditions (e.g., strong winds) could be favorable conditions as they can hinder the escape of fumes from chimneys.

Among the epidemiological criteria to be sought are the simultaneous involvement of several subjects of a group occupying a confined space, even with different clinics; all members of a family with flu symptoms but without fever; early onset of symptoms in small pets; the recurrence of flu-like symptoms, heart failure, and syncope; the diminution of symptoms outside a specific environment; suspect even in the case of a drunk person found unconscious in a car [4, 8, 9].

### Evaluation of signs and symptoms

The cornerstones of the diagnosis will be evaluation of signs and symptoms, the medical history, the values of carboxyhemoglobin from venous/arterial blood gas analysis (the reading of the handheld CO-oximeter must be compared with the latter), seasonality, evaluate the possible sources of monoxide production (to be searched with the eye and investigated).

The clinical scene in which carbon monoxide poisoning occurs is extremely varied in terms of symptoms and severity, as well as totally non-specific. These two aspects have earned carbon monoxide the names of “silent killer” and “great imitator.” One study showed that 3–5% of patients who presented to the emergency room had covert exposure to carbon monoxide not recognized at scene [8]. The frequency is higher in the cold season as the toxic gas is often produced by malfunctioning or inadequate heating means (e.g., braziers in closed places, and stoves). For this reason, the scene of intoxication is often the home. The variety of presenting signs and symptoms of carbon monoxide intoxication is listed in Table 1.

**Table 1:** Signs and symptoms in carbon monoxide poisoning [7, 8, 10]

***Central nervous system:*** headache (90%), altered conscious level (30%), lethargy (50%), dizziness, psychic alterations, sensory blunting and drowsiness, loss of consciousness, coma, tinnitus, ataxia, seizures, visual disturbances	
***Cardiovascular system:*** tachycardia, arrhythmias, angina or palpitations, signs of cardiac ischemia on ECG, acute pulmonary edema, cardiac arrest	
***Gastrointestinal system:*** nausea and vomiting (50%), diarrhea	
**Nonspecific:** malaise, subjective weakness (20%)	
***Other*:** rhabdomyolysis, lactic acidosis	

Seasonality, evaluation of the possible sources of monoxide production (to be searched with the eye and investigated) is listed in Table 2.

**Table 2:** Sources of exposure [4]

In domestic environment	e.g., stoves, boilers, chimneys, braciers
By engines left running in confined spaces	e.g., vehicles, lawnmowers, generators, pumps
In confined spaces	e.g., car interiors
In environments at risk	e.g., outboard speedboat, camion or pick up truck bed
In workplaces with possible environmental pollution	e.g., fireman, traffic wardens, garage workers, drivers, workers in tunnel

### Pre-hospital management

For the doctor, it is essential to apply what is a “privilege” of the first aid teams in the area, a rapid overview of the situation, and the possibility of observation and active research in the environment in which the rescue is carried out. The search for possible sources of intoxication, often in collaboration with other professional figures such as the Fire Brigade, is mandatory to avoid a failure to make a diagnosis and, indirectly, a failure to prevent further future cases of intoxication in the same environment. It should be emphasized that source detection is not always obvious or easily identifiable. Cases have been reported in which an established intoxication did not correspond to the identification of the gas production source [11]. In a study on carbon monoxide poisoning in hotels [12], it is highlighted how often the production of the toxic gas was not located directly in the vicinity of the room where the patients were staying, but could also be placed at a distance from some floors within the structure. Carbon monoxide (CO) also manages to spread through plasterboard walls [13]. The economic class of private or receptive accommodation was not a discriminant factor of greater or lesser probability of monoxide pollution, resulting in an indifferent variable. Sixty-five percent of the cases had occurred in structures belonging to nationally distributed hotel chains and there was no shortage of cases in luxury rooms. Since some accommodation facilities equip their environments with CO detectors in association with smoke detectors, it would be good to inquire about their presence in case of suspicion [14].

Knowledge of the “subtle” characteristics of this gas and the ability to assess the environmental scenario as far as possible are an essential aspect of the emergency clinician’s cultural background. In cases where the source of intoxication is not evident and is not sought, it is not always possible to refer the diagnosis to the emergency room doctor. He could be misled by the tendency of laboratory values to reduce/normalize themselves (mainly carboxyhemoglobin) due to transport times or possible normobaric oxygen therapy or by his own lack of concern for having detected high but not alarming levels of carboxyhemoglobin as in the case of a chronic intoxication which goes unrecognized.

**Fig. 1:**
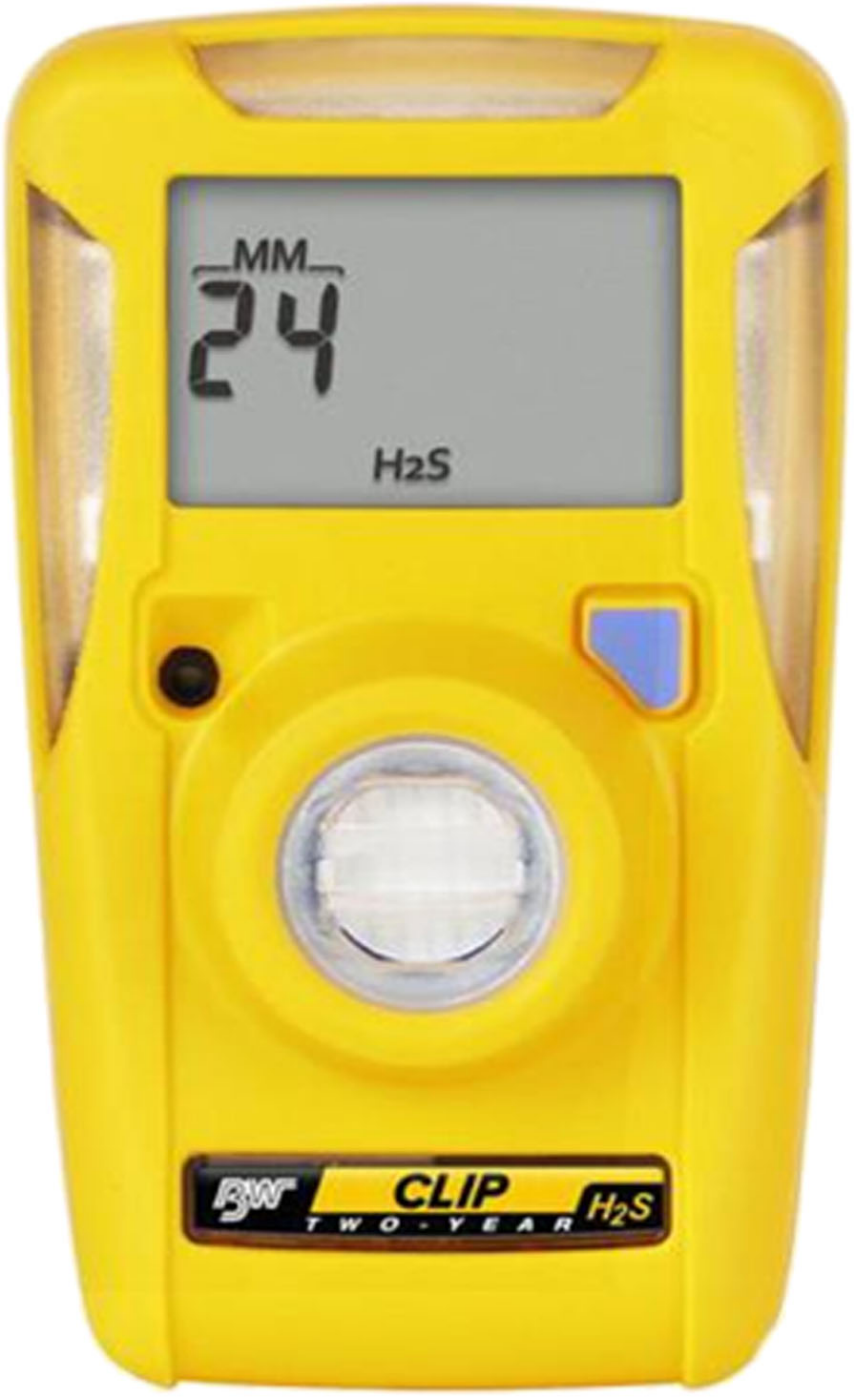
Example of a handheld environmental carbon monoxide detector

Many emergency teams are equipped with environmental detectors (Fig. 1) that measure the concentration of carbon monoxide in the air. This represents an undoubted advantage, considering that an audible alarm will be able to easily guide our search.

However, let us consider the possibility that on our arrival the patient has been removed from the room/closed place where he was when he suffered the malaise or lost consciousness. Furthermore, he himself may have moved independently from the contaminated area in which he was staying. In the physical space where the patient is visited, the detector therefore may not indicate anomalies in the composition of the air. The same thing can happen if windows or doors are opened at a later time. In these cases, the emergence of the suspicion of intoxication depends entirely on the “investigative” ability of the doctor and the means available to him in the area. Detection in an environment of abnormal levels of carbon monoxide (> 35 ppm) certainly facilitates the diagnosis but not all the emergency teams on the Italian territory are equipped with an environmental CO detector (whose malfunction, moreover, can feed the sources of error). In cases where the Fire Brigade is already present on the scene or intervene in support of the EMS, their instruments can compensate for any lack of equipment but this happens in a small percentage of cases.

### Common diagnostic errors

Due to the non-specificity of the symptoms and signs, great care must be taken in setting the differential diagnosis with other pathologies. The most frequently reported diagnostic errors in the literature are shown in Table 3. Among the most common errors are certainly the flu syndrome, non-specific gastrointestinal disorders, cold syndromes, further proof of the non-specificity of the clinical picture. In erroneous diagnoses, we must then include organic neurological pathologies, cerebral and cardio-vascular accidents, headaches, vertiginous syndromes, acute alcoholism-delirium tremens. Food poisoning can also represent a misdiagnosis, especially if the gastrointestinal symptoms are common to several people.

**Table 3:** Common diagnostic errors in carbon monoxide poisoning [4]

***Diagnostic errors***	***%***
Food poisoning	38
Psychiatric disorders (hysteria, confusion, depression)	18
Cardiac disorders with angina or syncope as presenting symptoms	13
Alcohol intoxication (which however can coexist and is an undoubted confounding factor) or delirium tremens	7
Acute solvent poisoning	7
Headache, migraine	6
Brain ischemic pathologies	4
Cerebral hemorrhage	4
Brain tumors (suspected in case of seizures)	3

A study that analyzed 3000 cases in a hospital in Catalonia reported as the most frequent misdiagnosis encephalitis, ischemic/hemorrhagic stroke, epilepsy, decompression pathology, tension headache/migraine, occupational conflicts, collective hysteria, food poisoning, and peripheral dizziness [3].

Inserting the differential diagnosis with carbon monoxide poisoning in all cases of suspected food poisoning or sudden malaise involving several people at the same time could be a sort of “safety formula” as the usual practice for the clinician [15].

There can be confusion related to the diversity of potentially toxic gases. Liquid gases derived from petroleum such as propane or butane do not contain carbon monoxide, but their inhalation can induce asphyxiation, not to be confused with carbon monoxide poisoning. If, on the other hand, there is also incomplete combustion of these gases, then CO is formed, which will lead to a mixed picture of asphyxiation and monoxide poisoning [3].

Chronic exposures to carbon monoxide seem far from infrequent and can represent a real pitfall for the clinician as they can give more nuanced symptoms as well as present low and unjustifiably reassuring values of carboxyhemoglobin (COHb).

As part of the differential diagnosis, it is also worth remembering the existence of acute endogenous carbon monoxide (CO) intoxication, resulting from the inhalation of pickling agents (methylene chloride) which lead to the formation of CO as a consequence of their hepatic metabolism [16]. Other causes of increased carboxyhemoglobin are hemolytic anemia (e.g., sickle cell anemia) and colon cancer [9, 17].

One of the elements that can make it more difficult to gather information about symptoms and circumstances is the presence of a language barrier. In the retrospective study by Maffi et al. conducted on 14 Italian hyperbaric centers, it was shown that 48.73% of the patients were not of Italian nationality [18].

The possible state of pregnancy of a patient must always be investigated and the lack of information about a state of pregnancy should be an unacceptable error. Fetal COHb levels are approximately 10–15% higher than maternal levels. Elimination is slower in the fetus. Maximal concentrations of CO in fetal blood are found after about 4 h [19].

There is not necessarily a correlation between the severity of the clinical presentation and the development of delayed neurological sequelae, which further complicates the initial classification and perspective view of the evolution of carbon monoxide intoxication disease [20].

### Errors related to the evaluation of carboxyhemoglobin (COHb) on blood sample

Carbon monoxide poisoning can be confirmed by the detection on a blood sample of a quantity of carboxyhemoglobin of at least 3–4% in the non-smoker, greater than 10% in the smoker (in general for each packet of cigarettes smoked per day, carboxyhemoglobin increases by approximately 2.5%; rarely in heavy smokers, especially with lung disease, it can exceed 10%) [21].

A good practice would be for the doctor at the scene to take an initial blood sample with a heparinized syringe. This would allow doctors to have blood carboxyhemoglobin data in the acute phase, which is important for the evaluation of the case and for therapy in a hospital setting or at the hyperbaric center [22]. This is a venous blood sample; there is no need for an arterial sample, since the arterial and venous vascular compartments quickly reach equilibrium due to the high diffusibility of carbon monoxide. This good practice is especially useful because in most cases, the measurement of blood carboxyhemoglobin takes place upon the patient’s arrival in the emergency room. He often arrives there after being subjected to normobaric oxygen therapy by emergency medicine staff. Carboxyhemoglobin (COHb) has half-life times that decrease with increasing inhaled oxygen concentration. In a healthy person with intact respiratory function, its half-life is 74 min by breathing 100% of normobaric oxygen (but in practice, there are several cases in which even after several hours the measured levels are higher than expected). Even the simple removal of the injured person from the polluted place will change the blood hemoglobin content linked to carbon monoxide (CO), reducing it. A half-life of 320 min is calculated if the patient is placed in an environment without sources of toxic gas. So what we will measure will depend on the time that has elapsed from leaving the scene, the administration or non-administration of normobaric oxygen and its duration, the FiO_2_ administered [4, 21, 23].

We should recall that the initial damage is of a hypoxemic nature, followed by a systemic inflammatory response and the involvement of mitochondrial respiration that persists even after the blood levels of carboxyhemoglobin have returned to normal. Furthermore, carbon monoxide binds not only to hemoglobin but also to many proteins, the degree of dysfunction of which has not yet been determined. In addition, tissue levels of monoxide are not measurable. Despite what has been said, it is proven that even when the available elements give almost certainty of exposure, some doctors accept the diagnosis of monoxide poisoning only if they find high COHb values, considering serious only cases in which they are far beyond the norm [3, 24].

The universally recognized value of the measurement of blood carboxyhemoglobin is only to confirm that the subject has come into contact with carbon monoxide in the previous hours, a sort of memory of the link between it and hemoglobin. The percentage obtained from this measurement will not be proportional to or correlated with severity or indicative of prognosis, especially in cases of chronic exposure. In fact, it is not uncommon for clinically mild intoxications to present elevated carboxyhemoglobin values while patients in coma have low values [3].

It is important to remember that chronic exposures can lead to some of the most serious sequelae, although often presenting with not markedly high levels of carboxyhemoglobin [24, 25].

In all cases in which combustion fumes have been inhaled (for example in a patient who has been burned or saved from a fire in a closed environment), carbon monoxide as well as cyanides intoxication must be suspected. It is useful to consider that the administration of the antidote for cyanides, hydroxocobalamin, can significantly alter the accuracy of the detection of COHb in the blood sample [19]. In our opinion, it is advisable to perform the test before proceeding with any therapy with the cyanides antidote.

### Errors related to the measurement of oxyhemoglobin

An important source of confusion is represented by the evaluation of arterial oxygenation both in its analysis on a blood sample with a spectrophotometric technique and in its evaluation with transillumination with a pulse oximeter. The most recent laboratory spectrophotometers, by transilluminating a blood sample with multiple wavelengths, directly calculate the concentrations of oxyhemoglobin, deoxyhemoglobin, carboxyhemoglobin, and methemoglobin, as they detect the specific absorption spectra of each of them. SaO_2_ (arterial oxygen saturation) measured will be a net value of all other forms of hemoglobin and so the carboxyhemoglobin will be measured directly by a CO-oximeter incorporated in the machine.

Older blood gas analyzers, on the other hand, do not contain a CO-oximeter but calculate SaO_2_ indirectly, using algorithms based on the hemoglobin dissociation curve and the effect of pH. Therefore, the arterial saturation of hemoglobin will also erroneously contain the amount of hemoglobin linked to carbon monoxide, which does not contribute to tissue oxygenation.

Another possible source of error concerns the standard pulse oximeters used for measuring SaO_2_. Those using 2 wavelengths (660 and 990 nm) cannot differentiate carboxyhemoglobin (COHb) from oxyhemoglobin (O_2_Hb) because COHb and O_2_Hb have similar absorption spectrum (extinction coefficient) at 660 nm and are therefore detected with a value of oxyhemoglobin representing their sum. In a study conducted on 30 people with COHb > 25%, 2-length pulse oximetry revealed a SaO_2_ in any case greater than 90% in all patients [21].

### Reducing margin of errors: quality and timing

**Fig. 2:**
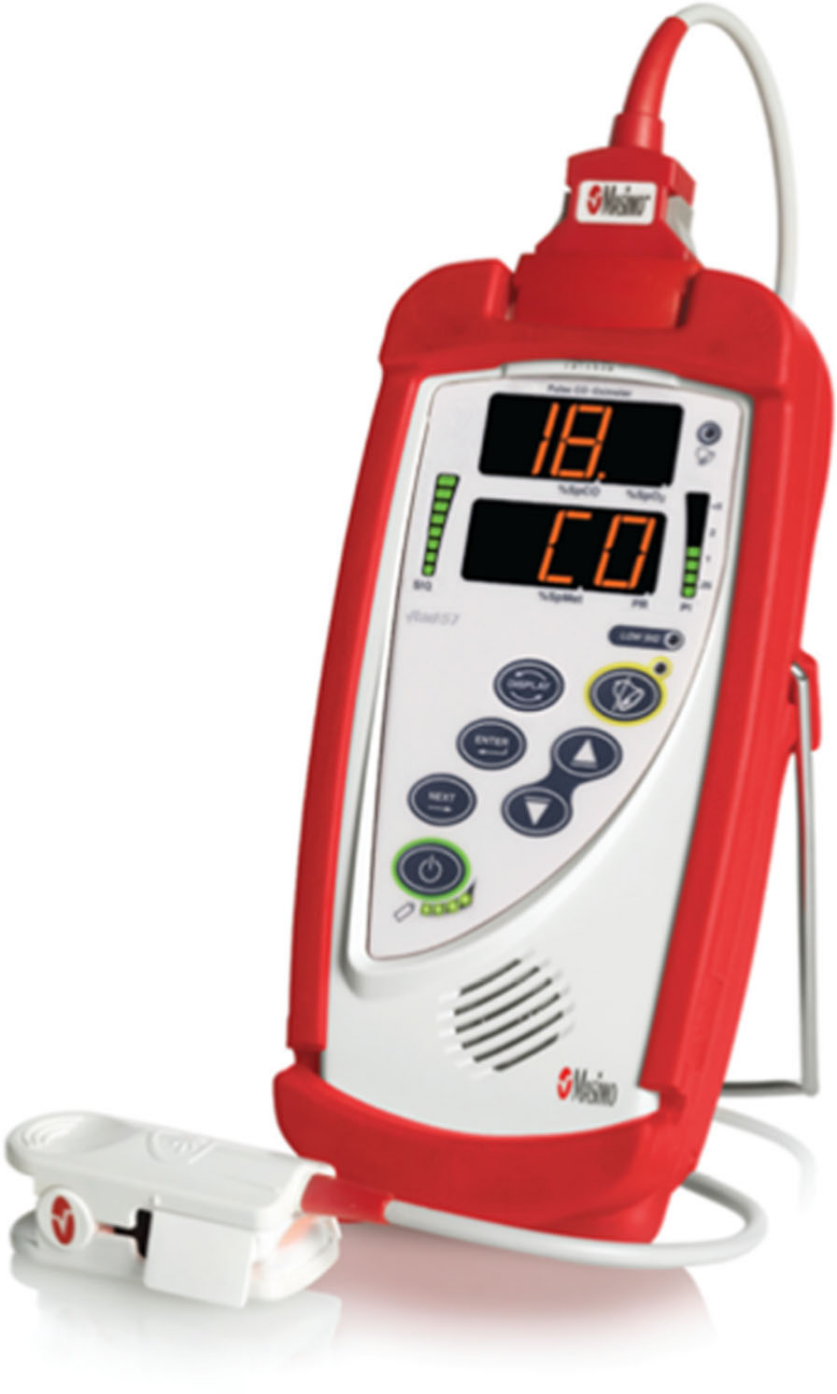
Masimo Rad 57 handheld CO-oximeter

Portable CO-oximeters, which have been available on the market since 2005, allow the non-invasive measurement of carboxyhemoglobin directly on the territory. In theory, they are capable of bringing to light unrecognized carbon monoxide poisoning, reducing the margin of error in the quality and timing of diagnosis [26] (Fig. 2).

Suner’s study published in 2008 reported the use of the portable CO-oximeter as a screening tool to proceed with the measurement of carboxyhemoglobin from a blood sample on patients in an emergency department regardless of the type of symptoms/cause of arrival. Out of 10,856 patients examined, he reported 11 people with non-specific signs/symptoms whose diagnosis was directed towards carbon monoxide poisoning thanks to the non-invasive meter [27]. To date, the reliability of the handheld CO-oximeter is not unanimously proven but it certainly contributes to reducing the risk of misdiagnosis. It is always recommended to perform a measurement of carboxyhemoglobin on a blood sample to confirm and give more valid data for the diagnosis. Thom and others advise to always perform this blood chemistry analysis before deciding on a possible transfer to the hyperbaric chamber [21].

One of the most used CO-oximeters, Rad 57, has among the specifications indicated by the manufacturer for the saturation of carboxyhemoglobin (% SpCO), an accuracy of 1–40 ± 3%, and a resolution of 1%; total hemoglobin saturation accuracy (% SpHb g/dl) of 8–17 ± 1 g/dl and a resolution of 0.1 g/dl.

It is good to know that the carboxyhemoglobin values shown by the instrument (SpCO) do not always correlate with those (COHb) measured on a blood sample. An overestimation of the SpCO compared to the COHb has been reported as more pronounced in the non-smoker than in the smoker [28]. In a study carried out on 1363 patients referred to the Emergency Department, 9% of false positives and 18% of false negatives were reported with respect to the cut-off value of diagnostic carboxyhemoglobin for intoxication [29].

The CO-oximeter is considered acceptably accurate in normoxia. However, it is not uncommon for a patient intoxicated by carbon monoxide to present hypoxemia, which is possible especially when there is simultaneous inhalation of fumes released by fires. In a study conducted on the Masimo Rainbow SET® Radical-7 handheld CO-oximeter model, it was found that in the presence of mild hypoxemia and a blood COHb of 10–12%, the handheld instrument maintained accuracy in detecting hypoxia. With a slight-moderate reduction in arterial oxygen saturation (SaO_2_), the accuracy of the non-invasive measurement of carboxyhemoglobin was not appreciably reduced, but the precision was slightly reduced. For SaO_2_ values < 85%, the handheld device did not provide the numerical value of carboxyhemoglobin, but only a standard abbreviation (“low signal IQ”) or a blank value could be displayed. For carboxyhemoglobin values close to zero, a blank value was sometimes displayed in the SpCO field. The reading of higher values of peripheral oxygen saturation (SpO_2_) compared to SaO_2_ was also reported [30]. In general, the most modern devices are handheld instruments capable of measuring SpCO, peripheral oxygen saturation (SpO_2_), methemoglobin (SpMet), SpOC (oxygen content), and PVI (plethysmographic variability index, useful for evaluating the response to fluid administration in mechanically ventilated patients and improving fluid management).

From these considerations, it is clear that even the use of a device such as the handheld CO-oximeter does not guarantee against evaluation errors but could itself become a source of error. According to Weaver, RAD 57 is not a valid tool for triage, nor should it lead to discarding the hypothesis of CO-related intoxication in suspected cases but it can be useful in cases with non-specific symptoms such as flu-like ones, in which the detection of a high carboxyhemoglobin level measured with a non-invasive method can positively orient our diagnosis. We should always take into account that the diagnosis of exposure to carbon monoxide is always essential for the evaluation of carboxyhemoglobin in blood [29].

It should be emphasized that not all the Emergency Medicine Services are equipped with a portable CO-oximeter but are often equipped with only the standard pulse oximeter for measuring SaO_2_.

The use of the CO-oximeter on the scene combines a greater speed in diagnosis with an organizational and economic advantage according to some authors. The ability to diagnose suspected cases of carbon monoxide exposure in a timely fashion. Detecting elevated COHb levels to enable rapid initiation of appropriate treatment, including normobaric and hyperbaric oxygen, may improve outcomes [31].

A clinical suspicion of intoxication made on the territory involves a blood dosage of COHb upon arrival in the emergency room. For those authors, using the instrument at home, we would avoid performing the blood dosage on all those patients for whom the set of clinical and situational assessments associated with normal values detected by the CO-oximeter would exclude the diagnosis of carbon monoxide poisoning [4]. Other methods have been proposed for the dosage of COHb in an emergency; they are evaluated to be not easy to apply in a scenario such as that of the rescue of patients with severe CO intoxication [32, 33].

### Overestimation of the utility of normobaric oxygen and ignorance of the late neurological syndrome

A possible error of the clinician may be the lack of knowledge of delayed neurological sequelae (DNS) that leads to inadequate attention being paid to immediate therapies aimed at preventing them [34, 35]. Studies have shown that DNS can develop in up to 45% of patients after acute CO poisoning [36]. The means available to us to prevent this is the use of normobaric and hyperbaric oxygen (where the latter is indicated). Some authors have proposed the administration of a neurological screening battery for emergency assessment to identify patients with subtle symptoms in low-level CO-poisoning or patients who require more aggressive therapy but the utility remains uncertain [37, 38].

Sometimes the administration of normobaric oxygen induces an improvement in the clinical presentation which, while not reflecting a real remission of the cascade of changes caused by poisoning, can lead the doctor to discard the hypothesis of referral to a hyperbaric chamber, ignoring the protocol of decision-making adopted in his Operating Unit. The doctor in charge of the rescue must therefore know how to frame the clinical scene and possible indications to the HBO_2_ treatment, referring to the main guidelines to make the treatment chain more fluid and effective, and this will involve the territorial emergency team, the emergency room doctor and the doctor in charge of the Hyperbaric Center. He will also be able to evaluate and report any contraindications to the hyperbaric environment [3, 24].

## Discussion

**Fig. 3:**
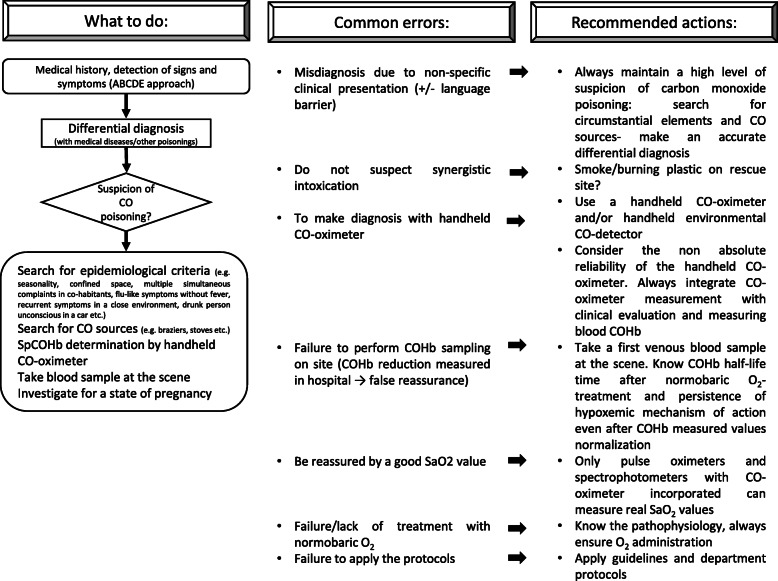
Practical tips for out-of-hospital management of carbon monoxide poisoning

Although carbon monoxide poisoning is a very frequent pathology, especially in the industrialized world, it is still a diagnostic challenge for the clinician today. This undoubtedly depends on the type of presentation of the clinical scenarios, which is so varied and non-specific; the complexity of the differential diagnosis with pathologies that have the same symptoms in common; the diversity in the instrumental equipment in the various emergency-urgency services; the subtle nature of the clinical course, which often with the removal from the source of carbon monoxide production sees improvements in the phase closest to acute but which can evolve into worsening in the long term. Perhaps the most important aspect to underline for the purpose of improving health care is that of gaps in knowledge. Today, although the study of the physiopathological dynamics underlying the syndrome is still in progress, we are able to define many of the cellular and systemic mechanisms responsible for acute and distant damage. Despite this, there are gaps in knowledge, or the emergency clinician is not updated on the subject. This contributes to a large extent to creating the diagnostic-therapeutic errors that we aim to investigate with this paper. Many experts emphasize the importance of always maintaining a high level of suspicion towards the possibility of being faced with an insidious case of carbon monoxide poisoning. It was highlighted that many correct and prompt diagnoses were influenced by the fact that an update on the subject had been put in place in the ward shortly before the clinical case was presented. The same cases of intoxication reported by national or local newspapers had contributed to raising the clinician’s sensitivity on the subject [10, 15]. Working groups for the drafting of intra-departmental guidelines or the in-depth study of an ever-current issue such as the one in question can contribute to the emergence of shared good practices. The same sharing of knowledge in verbal form or with the exchange of dissemination material among professionals is certainly a good and desirable thing to foster a virtuous circle that stimulates the curiosity of the doctor to conduct good health practices and possibly stimulate the adoption of evermore vanguard instruments. The use of simple and quick reference flow charts available in the ward can be helpful in obtaining an overview for the professional (Fig. 3).

## Conclusions

Ideally, carbon monoxide poisoning should no longer be an underdiagnosed syndrome or a poorly coded therapy, but a diagnostic challenge to be overcome. The doctor who deals with territorial emergency-urgency should feel intellectually stimulated in the search for environmental and clinical clues and naturally led to take an interest in the patient’s entire diagnostic-therapeutic process. This attitude will certainly be the best guarantee of the lowest possible risk of diagnostic and therapeutic errors and of the best outcome in terms of acute and long-term well-being for the patient.

## Data Availability

The data that support this study are available upon reasonable request.
